# Direct observation of negative-index microwave surface waves

**DOI:** 10.1038/srep22018

**Published:** 2016-02-23

**Authors:** J. A. Dockrey, S. A. R. Horsley, I. R. Hooper, J. R. Sambles, A. P. Hibbins

**Affiliations:** 1 Electromagnetic and Acoustic Materials Group, Department of Physics and Astronomy, University of Exeter, Stocker Road, Exeter, EX4 4QL, UK

## Abstract

Waves propagating in a negative-index material have wave-front propagation (wavevector, **k**) opposite in direction to that of energy flow (Poynting vector, **S**). Here we present an experimental realisation at microwave frequencies of an analogous surface wave phenomenon whereby a metasurface supports a surface mode that has two possible wavevector eigenstates within a narrow band of frequencies: one that supports surface waves with positive mode index, and another that supports surface waves with negative mode index. Phase sensitive measurements of the near-field of surface waves across the metasurface show the contrasting spatial evolution of the two eigenstates, providing a unique opportunity to directly observe the negative-index phenomenon.

Waves that propagate in any medium characterised by a negative refractive index are defined by the propagation of wave-fronts (*k*) that move in the opposite direction to the energy flow (*S*). Such behaviour was first reported in 1960 by Clarricoats and Waldron^1^ in circular metallic waveguides consisting of a high index core surrounded by lower index cladding. Given an injected wave with a known wavevector, the relative proportion of local energy flow in each medium will dictate the overall magnitude and direction of the Poynting vector. In a theoretical study in 1968, Veselago proposed^2^ that a similar result may be observed in a bulk material that possesses simultaneously negative electric permittivity ε and magnetic permeability μ. Such negative-index materials have not been found to exist in nature, so this idea lay dormant until the year 2000 when it was revived by Pendry^3^. Since then, a significant body of research has been devoted to developing artificial media (metamaterials) with negative refractive indices^4^,^5^,^6^, owing to a range of potential interesting possibilities such as a reversed Snell’s law^7^,^8^, a reversed Doppler shift^9^and a reversed Vavilov-Cerenkov effect^10^. It has also been shown that a reversed Snell’s law or ‘negative refraction’ can be observed using non-linear media, for example, four-wave mixing using graphene, as demonstrated by Harytyunyan *et al*.^11^.

One may extend the concept of a negative refractive index to other systems, such as electromagnetic waves bound to surfaces. An example of one such wave is the surface plasmon polariton (SPP)^12^; an electromagnetic surface wave that propagates non-radiatively along the interface between a metal and a dielectric and is typically found at optical and ultra-violet frequencies^13^, where there is significant field penetration into the metal. Indeed negative refraction of SPPs has been reported both in theory^14^ and in experiment^15^. In these examples, it is important to note that it is the surface mode index that is negative and not the bulk refractive index of the media either side of the interface. In bulk matter, a negative refractive index is often demonstrated by presenting a refracted signal on the ‘wrong’ side of the surface normal. However, the lack of phase sensitivity in such a bulk experiment does not readily allow for the relative directions of *S* and *k* to be resolved. It is the topic of this present study to provide detailed phase sensitive measurements of the local fields of microwave surface waves that evanescently decay from the interface to which they are confined, providing a unique opportunity to directly visualise the negative index phenomenon.

Due to the ease of experimentation and sample fabrication methods, we elect to undertake our studies in the microwave regime. At these low frequencies, metals are nearly perfectly conducting and SPPs are no longer strongly localised at the interface^16^: they are, in effect, simple surface currents. However, in 2004, Pendry showed theoretically^17^, and experiment later confirmed^18^, that perforating a planar metal surface with an array of sub-wavelength holes allows the surface to support SPP-like modes, even in the perfectly conducting limit. Although Pendry’s geometry is possibly the most well-known “spoof” SPP supporting ‘metasurface’^19^, there are a number of other well-documented geometries that give strong confinement of surface waves that are similarly analogous to SPPs^20^,^21^,^22^. Some of these were conceived many decades before the terms *metamaterial* and *spoof surface plasmon polariton* were coined, for example the grooved structures explored by Cutler in the 1940s (republished in 1994)^23^, developed in order to control the propagation of surface waves on the metallic skin of aircraft.

In this present work, we investigate a variation of the well-known ‘mushroom’ metasurface that was first proposed as a resonant high-impedance surface for microwaves by Sievenpiper *et al*.^24^. The transverse-magnetic (TM) surface mode supported by this metasurface exhibits ‘negative dispersion’^13^ allowing for a negative mode index to be achieved.

The dispersion curve for the TM surface mode supported by the sample studied in this paper is shown by the grey line in Fig. 1, where a schematic depicting a small section of this 45 cm × 45 cm sample is included as an inset. This dispersion curve is calculated using finite element method (FEM) numerical simulations (COMSOL Multiphysics). It is evident that, within a small frequency window (12.5 – 14.0 GHz), two wavevector eigenstates (*k*_+_ and *k*_–_) exist with contrasting signs of group velocity either side of the dispersion’s turning point. Since for low-loss systems, as studied here, the direction of the group velocity coincides with the direction of the energy flow^25^, this implies that one eigenstate supports surface waves with aligned *S* and *k* (positive mode index, denoted *k*_+_) and a second eigenstate supports surface waves with anti-aligned *S* and *k* (negative mode index, denoted *k*_–_). The dispersion curve is shown for both positive and negative *k*_||_, with the eigenstates that are denoted without a prime possessing a positive group velocity and those labelled with a prime possessing a negative group velocity. This is an important distinction, with the reasoning most easily explained if one considers this mode excited along the line (i.e. in 1D) to which this dispersion curve corresponds. In this case, it is these two pairs (primed and un-primed) of eigenstates that will contribute to separate interference patterns either side of a point source.

The presence of the turning point and the shape of the dispersion curve can be understood by examining the local power flow in two distinct regions of this metasurface. The magnitude of the integrated local power flow (also calculated from numerical simulations) above (red line) and below (blue line) the surface of the structure is also shown in Fig. 1 for positive *k*_||_. Unlike the surface waves supported by Pendry’s hole-array geometry^17^, surface modes supported by the mushroom array allow for power flow beneath the surface to which the mode is localised. Crucially, this power flows in the opposite direction to that in the air above the surface^26^. Hence the dispersion curve of the surface mode exhibits a maximum in frequency at a wavevector far from the Brillouin Zone boundary, where the magnitude of these contributions becomes equal; this is unlike the case for single interface SPPs on bulk metals where the magnitude of the power flow within the substrate cannot exceed that in the dielectric above^12^. Either side of this maximum, the dominant contribution to the power flow determines the net direction of the energy flux and hence, the sign of the group velocity.

The numerical calculations of the surface mode dispersion illustrated in Fig. 1 demonstrate the negative mode-index phenomenon along the ΓX direction in reciprocal space. However in order to fully understand the wave’s propagation on the surface, it is helpful to explore the full 2D band-structure (Fig. 2(A)). This data is re-plotted as iso-frequency contours (Fig. 2(B–F)), with the plot for 13.0 GHz in Fig. 2(D) corresponding to the plane drawn through the band structure in Fig. 2(A). In these diagrams, the red lines, labelled with outward-going arrows in Fig. 2(D), represent the *k*_+_ eigenstate contours with positive mode index, and the blue lines represent the *k*_–_ eigenstate contours (inward arrows) with negative mode index. In these plots, the *k*_–_ and *k*_+_ contours contain both the primed and un-primed eigenstates that are shown in Fig. 1, and so are simply referred to as the *k*_–_ and *k*_+_ contours. Although not a topic for study in this paper, it is also apparent that the contours for the two eigenstates merge above the frequency of the turning point of the mode’s dispersion mode in the ΓY direction. The appearance of the closed contours (purple lines) is due to the difference in this limiting frequency in the ΓX and ΓY directions, which itself is a consequence of Bragg scattering associated with the rectangular symmetry of the geometry.

In order to probe the surface eigenmodes supported by the mushroom metasurface (Fig. 1(A)), phase-sensitive near-field measurements are recorded using a Vector Network Analyser (VNA) and a motorised translation stage (see Fig. S1 in the Supplementary Material). A map of the measured instantaneous electric field profile^27^, recorded at 13.0 GHz for a centrally located point source is presented in Fig 3(A). Notice the presence of four narrow beams, and further notice the wavelength of the surface wave within these beams is much shorter than of the isotropic pattern on the bulk of the surface. By reviewing the iso-frequency contours at 13.0 GHz (Fig. 2(D)), the strongly anisotropic propagation characteristic of the *k*_–_ contour (blue) associated with the beams is clearly apparent. It is in stark contrast to the isotropic propagation of the *k*_+_ contour (red) responsible for the fields on the rest of the surface. Of course, the measured instantaneous electric field profile of surface waves emitted from a point source contains both of these contrasting excitation patterns, and although surface waves from both eigenstates are indeed present in all directions, the directional weighting of these two eigenstates vary drastically. Hence, along the beams the *k*_–_ eigenstate (negative mode-index) dominates the measurement, and outside of these beams, the *k*_+_ eigenstate (positive mode index) dominates the measurement.

There is a unique opportunity to *directly observe* the negative-index character of these surface waves if the instantaneous field plot (Fig. 3(A)) is animated in time (phase). This is beautifully visualised in the Supplementary Material in movie S1, where the wave-fronts within the beams (*k*_–_) move towards the source and the wave-fronts outside of the beams (*k*_+_) move away from the source. By construction, the experimental setup requires power to flow away from the source, and the fact that the wave-fronts within the beams are propagating in the opposite direction clearly demonstrates that the *S* and *k* vectors (i.e. the group and phase velocities) point in opposite directions, and that the system exhibits a negative mode index. This is entirely analogous to the propagation of waves in media with negative refractive indices, and is the first such experimental field map showcasing the negative-index phenomenon.

By interpolating the Cartesian grid of the measured phase map along the solid black lines shown in Fig. 3(A), a comparison between the phase cycles of surface waves with in-plane wavevectors defined by the *k*_+_ and *k*_–_ contours can be made. This analysis does of course make the assumption that only the surface waves with an in-plane wavevector defined as *k*_+_ are found outside of the beams and that only surface waves with an in-plane wavevector defined as *k*_–_ are found within the beams. The unwrapped (removing the 2π degeneracy) phase along these lines is shown in Fig. 3(B) (coloured dots), where it is evident that the phase for the two eigenstates cycles in opposite directions. The gradient of a least squares fit (coloured lines) to each of these data sets reveals the in-plane wavevector component 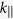, which is measured to be *k*_+_ = 327 ± 1 m^−1^ and *k*_–_ = −666 ± 2 m^−1^ along the relevant directions respectively. This compares favourably to the location of the peaks in the iso-frequency contour measurements (to be discussed shortly) along the relevant directions in Fig. 4(C) of *k*_+_ = 330 ± 16 m^−1^ and *k*_–_ = −662 ± 16 m^−1^.

By performing a 2D Fast Fourier Transform (FFT) on the derived instantaneous electric field measurements (see the methods section), the wavevectors, 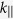 present in the measured field maps can be determined^28^. This technique therefore allows the eigenmodes present in the system to be mapped out, where these plots represent a convolution of the available eigenstates of the metasurface and the in-plane excitation pattern of the source. The measured iso-frequency contours are shown in Fig. 4, where the signals from the four quadrants have been averaged with respect to the symmetry planes of the metasurface, in order to improve the signal-to-noise ratio.

These measured FFT plots confirm the band structure of the eigenmodes supported by the sample and hence the iso-frequency contours. The peaks in the Fourier amplitude with larger in-plane wavevectors than that available to freely propagating plane-waves (white circles) are identified as the non-radiative surface wave solutions and the distribution of these peaks agrees very well with the modelled iso-frequency contours Fig. 2(B–F). By monitoring the positions of the peaks that correspond to the *k*_+_ and *k*_–_ contours, it is clear that they disperse in opposite directions. Thus the nature of the *k*_–_ eigenstate as having phase and group velocities of opposite sign, and of the *k*_+_ eigenstate as having phase and group velocities of the same sign is again confirmed. For a clearer demonstration of the evolution of the measured iso-frequency contours with frequency, see movie S2 in the Supplementary Material. These measurements are also reformatted in the form of a measured dispersion curve in supplementary Fig. S2, also in the Supplementary Material.

We have presented measurements demonstrating the flow of power (Poynting vector *S*) propagating in the opposite direction to the wave fronts (wavevector *k*) within beams of electromagnetic surface waves. This represents a surface-wave analogue of the negative-index phenomenon and these measurements constitute the first such direct experimental observation in the form of both a field map and iso-frequency contours that showcase this effect. We provide animations showing the phase-evolution of the generated surface waves in the Supplementary Material, as well as an animation of the evolution of the iso-frequency contours with frequency. The animated field map beautifully visualises the negative-index phenomenon since the wave-fronts within the beams propagate *towards* the source.

## Methods

### Sample fabrication

The metasurface investigated in this study is fabricated using printed circuit board (PCB) technology. First, an array of holes is drilled completely through a 0.79 mm thick, 450 × 600 mm panel of Nelco NY-9220^29^, clad either side with a 17 μm thick copper layer. Electroless deposition is subsequently used to deposit copper on the inner surface of the holes, where the outer radius of these hollow, metallic ‘pins’ is 150 μm. For the final step, the desired pattern of patches is etched into the top copper layer, so that a pin is located at the centre of each patch, providing electrical connection to the continuous lower copper layer (‘ground plane’).

### Experimental measurements

Our experimental methods allow for the measurement of the instantaneous electric field profile of the surface waves that are excited from point source located at the sample’s centre (an illustration is included in. Such a setup is preferable to other excitation mechanisms such as the Otto configuration^30^ since it is less invasive (for an Otto excitation, the close proximity of a high index dielectric to the sample surface will invariably alter the surface mode dispersion) and allows for the excitation of surface waves in all directions. Two antennas^31^ are used to provide the near-field excitation, and to probe the *z*-component of the local electric fields (see Fig. S1 in the Supplementary Material). They each comprise of a cut length of coaxial line, with the outer conductor and insulation removed from the final few millimetres, and are connected to separate ports of a vector network analyser (VNA) to provide measurements of both time averaged electric field magnitude and phase (continuous wave). The exact experimental arrangement involves one probe (that is designated as the source antenna) being threaded through a small hole that is drilled through the centre of the sample, at a point precisely between four patches. The corners of those four patches are slightly modified in order to prevent any electrical contact with the antenna. A second antenna (that is designated as the detector antenna) is oriented normal to and just above (~1 mm) the metasurface and is attached to a motorised *xyz*-stage. This antenna is scanned across an area of the sample of approximately 200 × 200 mm with a step size 0.675 mm in the *x* direction and 0.800 mm in the *y* direction (i.e. four measurement points per unit cell). The raw measurements of time averaged electric field magnitude |*E*_z_| and phase *φ* are subsequently combined to allow for the instantaneous electric field |*E*_z_| exp(i*φ*) to be derived^27^. A 2D Fast Fourier Transform (FFT) is then performed upon this data, with the magnitude of the Fourier terms indicative of the wavevector components present in the spatial field measurement. Since the metasurface has two orthogonal mirror symmetry planes, the signal-to-noise ratio in the FFT is improved by averaging the data from each of the four quadrants, hence imposing the symmetry of the metasurface onto the measurements.

An animation of the surface wave fields on resonance provides a beautiful demonstration of the negative mode index phenomenon. This animation (Movie S1) is generated by systematically advancing the phase (across a full temporal cycle), with the instantaneous electric field recalculated for every increment in phase. This allows the dynamics of the available surface-wave eigenstates to be directly visualised.

## Additional Information

**How to cite this article**: Dockrey, J. A. *et al*. Direct observation of negative-index microwave surface waves. *Sci. Rep.* **6**, 22018; doi: 10.1038/srep22018 (2016).

## Supplementary Material

Supplementary Information

Supplementary Information

Supplementary Information

## Figures and Tables

**Figure 1:**
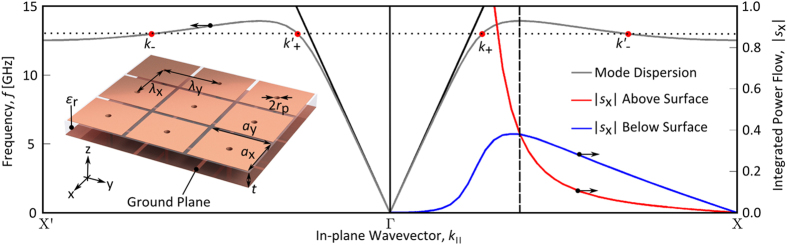
The rectangular ‘mushroom’ array investigated in this study supports a transverse-magnetic surface mode. Predictions of the dispersion curve for a surface mode along the Γ to X direction is shown (grey line), where two wavevector eigenstates (*k*_+_ and *k*_–_) exist within a small frequency window. The eigenstate labelled as *k*_–_ has opposing *S* and *k* as a consequence of the magnitude of the integrated local power flow below the surface (where it is negative, blue line) exceeding that above the surface (where it is positive, red line). The eigenstates denoted with a prime have a negative group velocity and those that are not have a positive group velocity. (Inset) A schematic depicting a small section of the ‘mushroom’ sample, that consists of an array of 17 μm thick rectangular copper patches of size *a*_x_ = 2.47 mm × *a*_y_ = 2.97 mm, arranged in a rectangular lattice of pitch *λ*_x_ = 2.70 mm × *λ*_y_ = 3.20 mm, where each patch is separated from a ground plane by a *t* = 0.79 mm thick dielectric (*ε*_r_ = 2.220 + 0.002i) layer, but electrically connected to it by a centrally located pin of radius *r*_p_ = 0.15 mm that is located at the centre of each patch.

**Figure 2:**
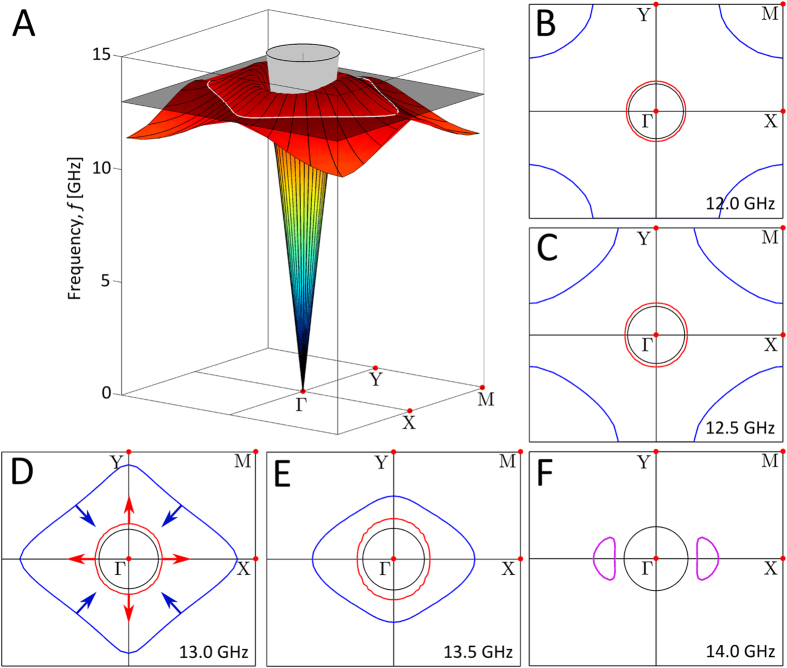
The surface wave eigenstates for the rectangular ‘mushroom’ array, calculated using numerical simulations. (**A**) The dispersion surface of the mode where the colour represents the frequency. The grey cone represents the ‘light cone’, the three dimensional representation of the light circles, and the dark transparent plane drawn at 13.0 GHz demonstrates how the dispersion surface corresponds to the iso-frequency contour plots (**D**). (**B–F**) The iso-frequency contours for this surface mode are shown for several frequencies, where the *k*_+_ eigenstate is signified by the red lines and the *k*_–_ eigenstate is shown by the blue lines. Purple lines indicate iso-frequency contours for frequencies above the surface mode limit frequency along the ΓY direction, where the *k*_+_ and *k*_–_ contours merge and create two closed contours. The black circles (‘light circles’) represent the maximum in-plane wavevector obtainable for a freely propagating plane-wave at each frequency and the coloured arrows represent the direction of group velocity *v*_*g*_ = ∇_*k*_*ω*, for the relevant eigenstate; the latter also highlighting that the two contours disperse in opposite directions as frequency is increased.

**Figure 3:**
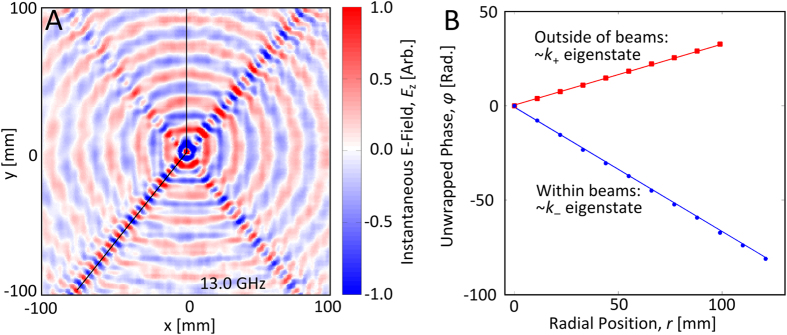
Measurements of surface waves emitted from a centrally located point source on the rectangular ‘mushroom’ array metasurface. (**A**) The measured instantaneous electric field (*E*_z_) map for a frequency of 13.0 GHz, where the source antenna is located at the centre of the plot. The point source excitation pattern for the *k*_+_ eigenstate is almost perfectly isotropic whereas the excitation pattern for the *k*_–_ eigenstate comprises of four beams and so the measurement represents a convolution of both. The variation between the magnitudes of the four beams is a consequence of experimental inaccuracies in the form of imperfect azimuthal invariance of the excitation pattern of surface waves emitted from the source and the angles from which the beams propagate outwards from the source is representative of the rectangular symmetry of the sample. (**B**) By interpolating the Cartesian grid of the measured phase map along the solid black lines shown in (**A**), a comparison between the phase of the *k*_+_ and *k*_–_ eigenstates can be made. The unwrapped (removing the 2π degeneracy) phase from outside of the beams (*k*_+_ eigenstate) is shown by the red dots and the unwrapped phase from within a beam (*k*_–_ eigenstate) is shown by the blue dots, with the coloured lines representing least squares fits. It is evident that the phase along these two lines cycles in different directions, with the negative gradient of the unwrapped phase within the beam implying the presence of a negative mode index.

**Figure 4:**
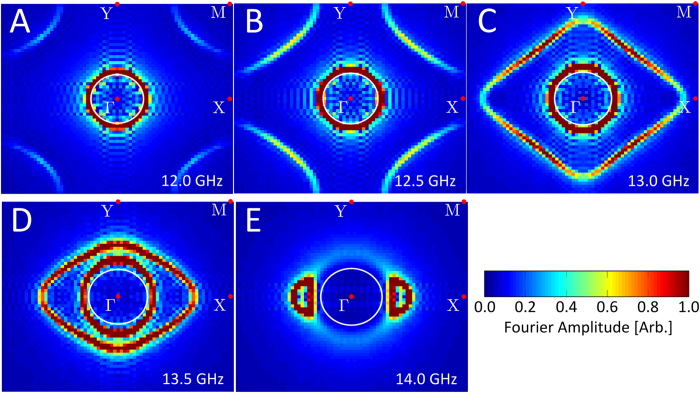
Measurements of the iso-frequency contours of the surface mode supported by the rectangular ‘mushroom’ array metasurface. The magnitude of 2D Fast Fourier Transforms (FFT) of the measured instantaneous electric field maps such as that shown in Fig. 3(A) is shown for a series of frequencies, where the ‘peaks’ in Fourier amplitude (colour scale) correspond to the iso-frequency contours shown in Fig. 2. The signals from the four quadrants of the FFT have been averaged with respect to the symmetry planes of the metasurface in order to improve the signal-to-noise ratio (the Supplementary Material details the calculation of these plots) whilst the white circles represent the ‘light circles’ at the various frequencies.
