# Supplementary figures and images for: Direct observation of negative-index microwave surface waves
Source: Sci Rep. 2016 Feb 23;6:22018. doi: 10.1038/srep22018 (PMC4763249; doi:10.1038/srep22018)

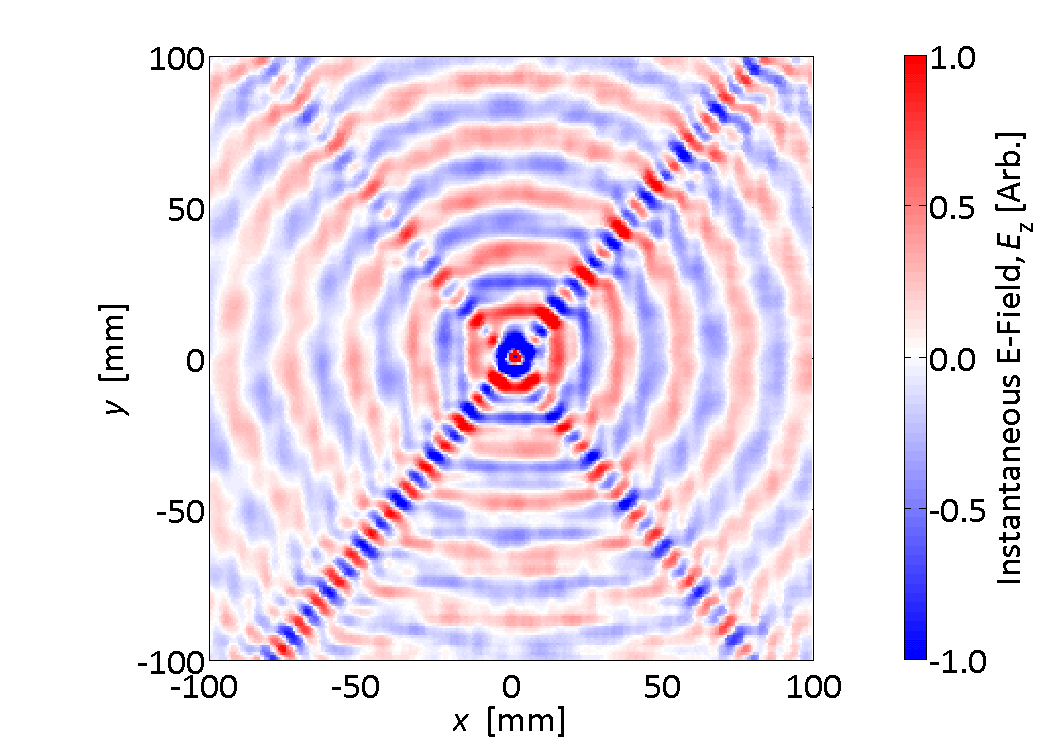

Supplement: Supplementary Information [file srep22018-s1.gif]

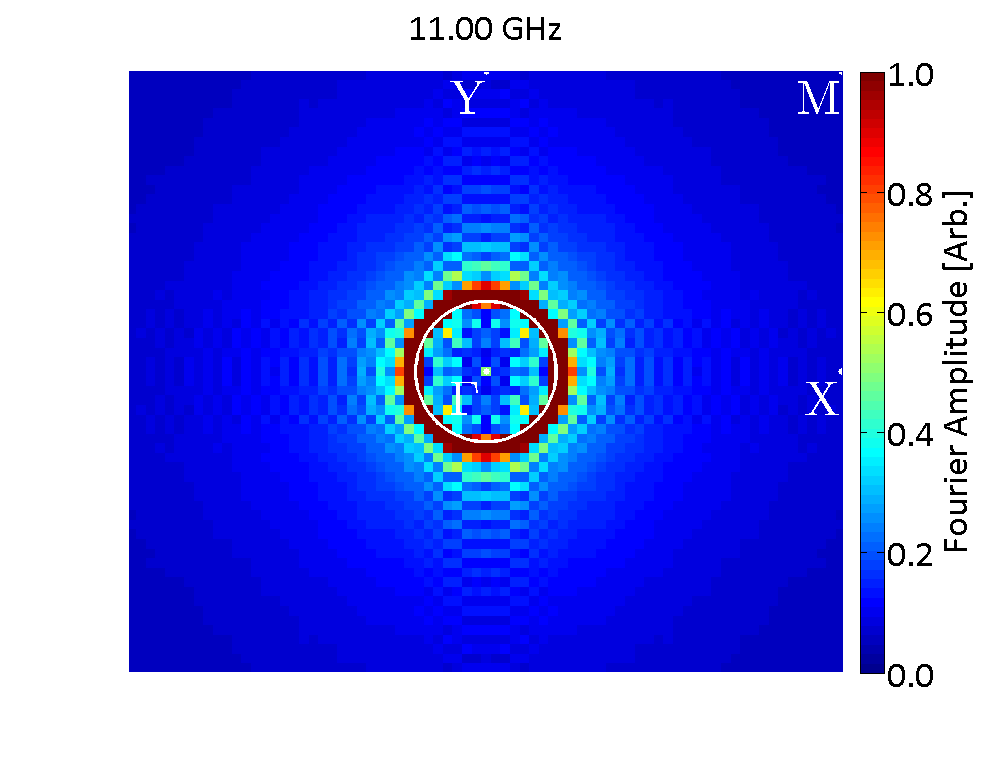

Supplement: Supplementary Information [file srep22018-s2.gif]
