# Supplementary material for: Direct observation of negative-index microwave surface waves
Source: Sci Rep. 2016 Feb 23;6:22018. doi: 10.1038/srep22018 (PMC4763249; doi:10.1038/srep22018)
Supplement: Supplementary Information [file srep22018-s3.pdf]

Direct observation of negative-index microwave surface waves

J. A. Dockrey, S. A. R. Horsley, I. R. Hooper, J. R. Sambles, A. P. Hibbins

Electromagnetic and Acoustic Materials Group, Department of Physics and Astronomy, University of Exeter, Stocker Road, Exeter, EX4 4QL, UK

Supplementary Material

An illustration of the experimental arrangement

For completeness, an illustration of the experimental arrangement is included as Fig. S1.

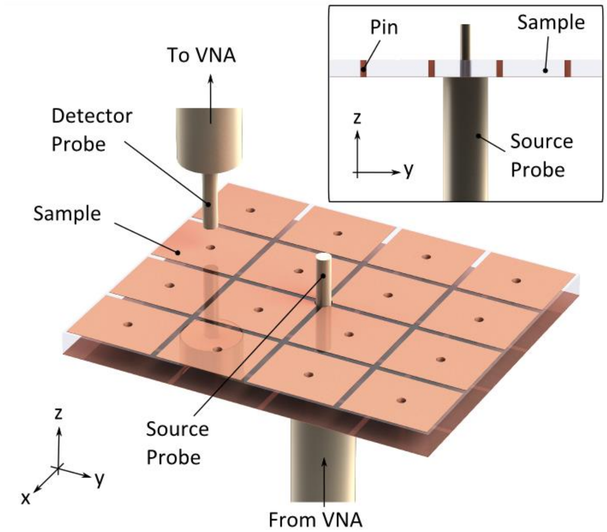

Figure S1 | The experimental arrangement. Surface wave excitation and detection is achieved using a pair of near-field antennas. The source probe is inserted through a small hole in the sample and fed with a signal from the VNA. The detector probe is held a small distance (~1 mm) above the sample in the opposite orientation feeding its measured signal back to the VNA, allowing for measurements of both magnitude and phase of the near-fields of surface waves. In this schematic, it is only a small section of the sample used in this study that is shown (see Fig. 1 for dimensions).

Dispersion curve measurement

By calculating the magnitude of the Fourier components from the measured instantaneous electric field maps, along the directions defined by the points of high symmetry (i.e. the Fourier amplitude between the Γ, X, M, Y and Γ points in sequence from Fig. 4) for a range of frequencies, a dispersion curve of the supported modes is generated (Fig. S2).

This dispersion curve clearly shows that, in the frequency range of approximately 11.4 GHz – 14.2 GHz, two unique surface wave eigenstates exist (k_- and k_+) for a given frequency. These eigenstates disperse in opposite directions with changing frequency, with the k_- eigenstate clearly reducing in wavevector with increasing frequency. This region of negative mode index (or 'negative dispersion') is indicative of a 'left-handed' (or 'backward wave' [1]) mode that shares propagation characteristics with plane waves in bulk negative index media; the phase flow and energy flux point in opposite directions. The amplitude of the Fourier components reduces in the directions defined as X to M and Y to M because for these wavevectors, the wave becomes so tightly confined to the metasurface that the overlap of the fields of the surface mode with the fields of the probe reduces drastically. However, the shape of the dispersion curve clearly possesses a turning point, where the group velocity must be zero. A Lifschitz tail [S2] is clearly visible close to this point of minimal group velocity, associated with the van-Hove singularity [S3] (i.e. a large photonic density of states).

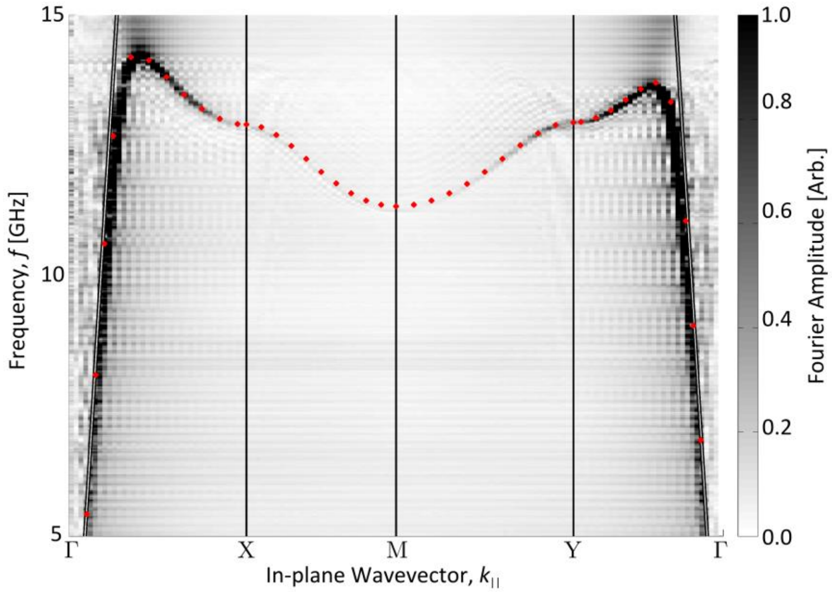

Figure S2 | The measured dispersion curve for the surface mode supported by the rectangular 'mushroom' array. The Fast Fourier Transform plots shown in Fig. 4 can be reformatted as a dispersion curve (grey scale) and compared to the predictions from full wave numerical simulations (red dots). Regions of negative mode index are clearly evident in the frequency range 11.4 GHz – 14.2 GHz, with good agreement between the experiment and model.

A short-pitch beating phenomenon

An astute observer may notice a short spatial-period interference phenomenon in the instantaneous electric field measurement (13 GHz, Fig. 3(A)) that is most clearly visible along any of the four beams. This is shown with increased clarity in the unprocessed time-averaged electric-field magnitude measurement in Fig. S3. Along the beams, an interference pattern can be seen that has a periodicity of ~ 6.3 mm. This is simply beating between the k_+ and k_- surface wave eigenstates (or the k'_+ and k'_- eigenstates see Fig. 1). The k_+ eigenstate is almost perfectly isotropic and so it can be assumed that with an isotropic point source, an isotropic excitation pattern is expected. However as mentioned in the main text, the four linear regions of the k_- eigenstate lead to an excitation pattern that almost exclusively consists of four beams. Hence, along the direction of these beams, two surface wave signals are indeed present, albeit with different amplitudes.

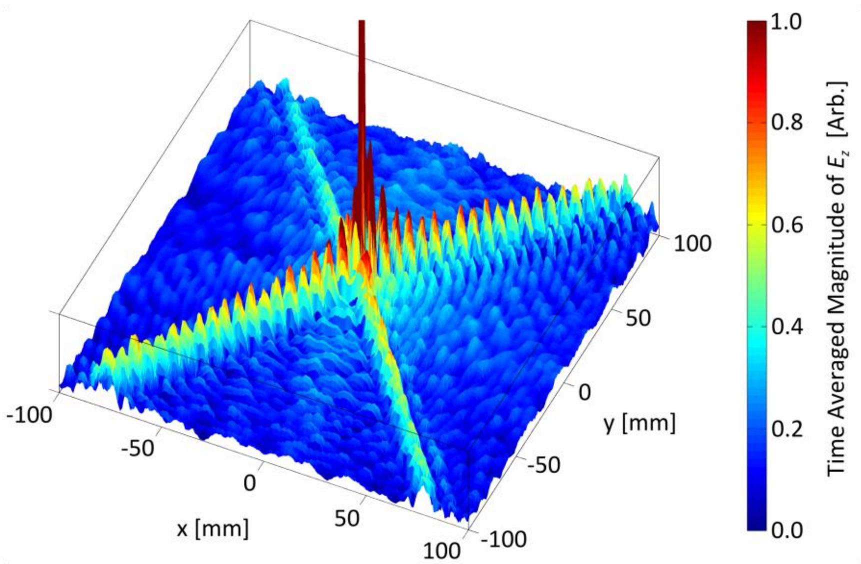

Figure S3 | Measured time averaged electric field map at 13.0 GHz. The time averaged electric field data that is used to determine the instantaneous electric field map shown in Fig. 3(A). Short-period beating between surface waves with in-plane wavevectors defined as k_+ and k_- (or k'_+ and k'_- see Fig. 1) is clearly visible along each of the four beams that is a consequence of the opposite sign of the wavevectors for the two eigenstates. Since the experiment insists on a positive energy flux (from the point source), only solutions with positive group velocity can be considered. Hence any short pitch beating is evidence for the 'left-handed' character of the k_- eigenstate, because to achieve this short wavelength, large wavevector beating, the two components must have opposite signs of phase velocity.

Fig. 3(B) shows the raw phase data after it has been unwrapped along the directions defined by the black lines in Fig. 3(A), using the built-in unwrap algorithm in MATLAB [S1]. Since the measured data is in the form of a Cartesian grid, a 2D interpolation algorithm ('interp2' [S1]) is used to allow the measured phase at the required coordinates to be determined. Then by taking the linear gradient of a plot of unwrapped phase as a function of radial distance from the point of excitation, the wavevector can be determined (the error in this fit is given as the error in this derived value of $k_{||}$). These values are directly comparable to the values determined from the FFT, where the error in the latter is given as the resolution in *k*-space. Note that the gradient of the straight line fit to the unwrapped phase as a function of distance gives the wavevector of the stronger of the two signals.

Movie S1

Movie S1 shows the propagation of surface waves at a frequency of 13 GHz (Fig. 3(A) animated temporally). The contrast between the point source excitation patterns for the k_+ and k_- surface wave eigenstates splits the measured instantaneous electric field map into two regions; one where although both eigenstates are present, the k_- eigenstate is excited more strongly (the four beams) and a second where the k_+ eigenstate is almost exclusively present (everywhere else). This temporal animation allows for a *direct visualisation* of the electric fields of propagating surface waves that are entirely analogous to waves that propagate in negative index media. Although the wave fronts associated with k_+ propagate *away from* the source, the wave fronts associated with the k_- eigenstate (within the beams) propagate *toards* the source. It is this difference that conclusively showcases the negative index phenomenon.

Movie S2

Movie S2 shows the evolution of the measured iso-frequency contours in the frequency range of 11.0 GHz – 14.2 GHz. The scale is renormalized for every frequency, using the mean of the instantaneous electric field magnitude at that frequency at all measured positions.

References and Notes

[S1] MATLAB and Statistics Toolbox Release 2012b, The MathWorks, Inc.

[S2] Huisman, S. *et al.* Measurement of a Band-Edge Tail in the Density of States of a Photonic-Crystal Waveguide. Phys. Rev. B, **86** 155154 (2012).

[S3] Hove, L. V. The Occurrence of Singularities in the Elastic Frequency Distribution of a Crystal. *Phys. Rev.,* **89**, 1189 (1953).
